# Mutation of *OsGIGANTEA* Leads to Enhanced Tolerance to Polyethylene Glycol-Generated Osmotic Stress in Rice

**DOI:** 10.3389/fpls.2016.00465

**Published:** 2016-04-18

**Authors:** Shuai Li, Wenhao Yue, Min Wang, Wenmin Qiu, Lian Zhou, Huixia Shou

**Affiliations:** ^1^State Key Laboratory of Plant Physiology and Biochemistry, College of Life Sciences, Zhejiang UniversityHangzhou, China; ^2^College of Life Sciences, Qingdao Agricultural UniversityQingdao, China

**Keywords:** GIGANTEA, osmotic stress, water deficiency, stomata, rice

## Abstract

Water deficit is one of the most important environmental stresses limiting plant growth and crop yield. While the identification of many key factors involved in the plant water deficit response has greatly increased our knowledge about the regulation system, the mechanisms underlying dehydration tolerance in plants are still not well understood. In our current study, we investigated the roles of the key flowering time regulator, OsGIGANTEA (OsGI), in the osmotic stress tolerance in rice. Results showed that mutation of *OsGI* conferred tolerance to osmotic stress generated by polyethylene glycol (PEG), increased proline and sucrose contents, and accelerated stomata movement. In addition, qRT-PCR and microarray analysis revealed that the transcript abundance of some osmotic stress response genes, such as *OsDREB1E, OsAP37, OsAP59, OsLIP9, OsLEA3, OsRAB16A*, and *OsSalT*, was significantly higher in *osgi* than in WT plants, suggesting that OsGI might be a negative regulator in the osmotic stress response in rice.

## Introduction

Water availability is a critical environmental factor for plant growth and development. To cope with water shortages, plants have developed multiple mechanisms to preserve cellular water homeostasis, including morphological, physiological and biochemical modulations that enhance water uptake and reduce water loss (Hincha et al., 2002; Chaves et al., 2003; Villadsen et al., 2005; Valliyodan and Nguyen, 2006; Hadiarto and Tran, 2011). A promising approach to enhance plant tolerance to dehydration stresses is the modulation of genes responsive to water deficiency (Yamaguchi-Shinozaki et al., 1995; Shinozaki et al., 1998). In recent years, several instances of crosstalk between the osmotic stress response and other signaling pathways, such as abscisic acid (ABA) signaling and flowering time regulation, have been identified (Ikegami et al., 2009; Fujita et al., 2011). For example, genes involved in flowering time regulation, such as *phytochrome B* (*phyB*) and *timing of CAB expression 1* (*toc1*), have been shown to be negative regulators in the response to dehydration conditions (Legnaioli et al., 2009; Liu et al., 2012).

GIGANTEA (GI) is regarded as a key component of flowering time regulation in many plant species (Fowler et al., 1999; Hayama et al., 2003; Hecht et al., 2007; Higuchi et al., 2011). In Arabidopsis, GI, CONSTANS (CO), and FLOWERING LOCUS T (FT) control photoperiodic flowering responses (Fowler et al., 1999; Mouradov et al., 2002; Srikanth and Schmid, 2011). Overexpression of *GI* promotes early flowering while *GI* mutants develop a large rosette of leaves and “gigantic” size under long day conditions due to a prolonged vegetative growth phase (Koornneef et al., 1991; Fowler et al., 1999; Park et al., 1999). The rice homolog of *GI, OsGI*, is also a circadian gene controlling diurnal rhythms of the global transcriptome and carbohydrate metabolism (Izawa et al., 2011), and mutation of *OsGI* causes late flowering under short day condition (Hayama et al., 2003; Izawa et al., 2011).

Besides its role in the regulation of flowering time and circadian rhythms, *GI* is also involved in processes such as sucrose signaling, starch accumulation and stress tolerance (Kurepa et al., 1998; Fowler and Thomashow, 2002; Dalchau et al., 2011; Kim et al., 2013; Riboni et al., 2013; Mishra and Panigrahi, 2015). In the past decade, *GI* has been shown to function in the response to several abiotic stressors including cold, salt, drought and oxidative stresses (Kurepa et al., 1998; Cao et al., 2005; Kim et al., 2013; Riboni et al., 2013). Expression of Arabidopsis *GI* was induced 5–8 fold by cold stress (Fowler and Thomashow, 2002). *GI* was proposed to regulate cold acclimation through a C-repeat Binding proteins (CBFs) independent pathway (Cao et al., 2005). *GI* mutants displayed increased cold sensitivity because the protective role of *GI* in cold tolerance was reduced (Cao et al., 2005). Recent evidence suggests that *GI* is a negative regulator for the tolerance to salt stress (Kim et al., 2013). Under normal conditions, Arabidopsis GI interacts with salt overly sensitive 2 (SOS2) to prevent SOS2-mediated SOS1 phosphorylation and activation (Kim et al., 2013). Under salt stress condition, GI is degraded and the freed SOS2 can interact with the Ca^2+^-activated sensor of sodium ions, SOS3, to activate and stabilize SOS1 (Kim et al., 2013; Park et al., 2013). Therefore, the *gi* mutant confers enhanced salt tolerance due to the constitutive activation of SOS1 (Shi et al., 2000; Kim et al., 2013; Park et al., 2013).

In addition, *GI* has been shown to regulate the response to oxidative stress which increases *GI* abundance and promotes flowering (Qian et al., 2014). *GI* mutants exhibit increased activation of superoxide dismutase (SOD) and ascorbate peroxidase (APX) and tolerance to a redox cycling agent, parquat, and H_2_O_2_ (Kurepa et al., 1998; Cao et al., 2006). Furthermore, it was recently discovered that *GI* can lead to early flowering and drought tolerance via the abscisic acid (ABA)-dependent activation of florigens under long day condition (Riboni et al., 2013).

Despite the increasing knowledge of GI's role in Arabidopsis, little is known about the biochemical and molecular functions of its rice homolog, *OsGI*, in response to water deficits. In this study, we investigated the role of *OsGI* in osmotic stress in rice. We determined that mutation of *OsGI* confers tolerance to osmotic stress generated by polyethylene glycol (PEG). Mutation of *OsGI* results in increased proline and sucrose contents and more rapid stomata movement. In addition, transcription analysis revealed that the expression of many genes involved in drought response is altered in *osgi* plants.

## Materials and methods

### Plant materials, growth conditions, and stress treatments

The rice (*Oryza sativa*) *osgi* mutant, *osgi*, was obtained from the Rice *Tos17* insertion mutant database at the Rice Genome Resource Center, Japan. *Osgi* and its wild type control, cv. *Nipponbare*, were used for all physiological experiments. For complementation of the *osgi* mutant, the *OsGI* coding sequence was expressed in a modified pCAMBIA1300 vector under control of the Cauliflower Mosaic virus 35S promoter (Wang et al., 2009). The construct was introduced into *osgi* mutants using a *Agrobacterium tumefaciens*-mediated transformation method (Wang et al., 2009). A modified culture medium containing 1.425 mM NH_4_NO_3_, 0.323 mM NaH_2_PO_4_, 0.513 mM K_2_SO_4_, 1.643 mM MgSO_4_, 0.998 mM CaCl_2_, 0.125 mM EDTA-Fe(II), 0.075 μM (NH_4_)_6_Mo7O_24_, 0.25 mM NaSiO_3_, 0.009 mM MnCl_2_, 0.019 μM H_3_BO_3_, 0.155 μM CuSO_4_, and 0.152 μM ZnSO_4_was used at pH 5.5 for hydroponic experiments(Yoshida et al., 1976; Li et al., 2014). Rice plants were grown in a growth room with a 12 h light/12 h dark cycle, a daytime temperature of 30°C and nighttime temperature of 22°C.

Rice seeds were first germinated in tap water for 2 days before being transferred into the culture media. For osmotic stress tolerance experiments, seedlings were transferred to culture media containing 21% polyethylene glycol (PEG) 8000. For gene expression analysis, plants were transferred to culture media containing 18% PEG 8000. For microarrays, leaves from 14-day-old *osgi* and WT seedlings grown under normal growth condition were used.

### Measurement of proline and sucrose contents

Proline content was measured as previously described (Bates et al., 1973). Briefly, approximately 50 mg fresh leaves from 15-day-old rice plants grown in hydroponics were harvested for analysis. Samples were homogenized in 5 ml 3% sulfosalicylic acid, incubated at 100°C for 10 min and pelleted by centrifugation. The resulting supernatant was collected, mixed 1:1:1 (2 mL each) with glacial acetic acid and acidic ninhydrin, and incubated at 100°C for 30 min. The chromophore was toluene extracted, and absorption values for the solution were detected at 520 nm wavelength. Proline concentration was determined using a standard concentration curve and adjusted to the fresh weight of leaves.

For sucrose analysis, approximately 200 mg fresh leaves were homogenized in 2 ml deionized water and centrifuged at 4°C for 10 min. The resulting supernatant was incubated at 100°C for 3 min, and sucrose content was determined using ion chromatography (ICS-3000, DIONEX).

### RNA preparation, quantitative real-time PCR

For microarray, total RNA was extracted from plant samples using RNeasy mini kits (Qiagen, USA, http://www.qiagen.com) according to the manufacturer's instructions. For quantitative reverse transcription PCR (qRT-PCR), total RNA was extracted from leaf samples using TRIzol Reagent (Invitrogen, CA, USA) according to the manufacturer's instructions.

First-strand cDNAs were synthesized from 4 μg total RNA using SuperScript II reverse transcriptase (Invitrogen), and qRT-PCR was performed using LightCycler 480 SYBR Green I Master Kit (Roche Diagnostics, USA) on a LightCycler480 thermocycler. The amplification steps and quantitative analysis of relative expression levels was performed as previously described (Li et al., 2014). RNA samples were collected from three biological replicates. Each sample was analyzed using three biological replicates and normalized to the housekeeping gene *OsACTIN*. All primers used for qRT-PCR analyses are listed in Supplementary Table 1.

### Microarray analyses

For the microarray analysis, leaves were sampled from plants grown for 14 days after germination (DAG) under normal condition. Genes were considered significantly differentially expressed when *p* < 0.05 and Posterior Probability of Differential Expression (PPDE) >0.95 with a 2 fold cut-off to correct for false discovery rates (Dalchau et al., 2011). Differentially expressed genes (up- or down-regulated) between *osgi* and WT were compared to a previously published microarray (Jain et al., 2007). The raw microarray data files have been supplied as Supplementary File 2.

### Stomata conductance (g_s_) and transpiration rate (T_r_) measurements

Stomata conductance (g_s_) and transpiration rate (T_r_) were recorded in *osgi* and WT plants inside a growth chamber on fully expanded leaves using an Li-6400 portable gas-exchange system (LI-COR) according to the manufacturer's instructions. All measurements were conducted 9 h after lights-on in saturating light (1500 μmol m^−2^ s^−1^) with 400 μmol mol^−1^ CO_2_ surrounding the leaf. Leaf temperature for all measurements was approximately 30°C (ambient temperature), and the leaf-to-air vapor pressure deficit (VPD) was kept abetween 1.5–2.5 kPa. To measure stomata movement in response to PEG treatment, *osgi* and WT plants were grown in hydroponics under normal condition for 5 weeks and then treated with 18% PEG 8000 at 4, 6, and 8 h after lights-on.

### Statistical analysis

Statistical significance was determined using the SAS program with *t*-test (SAS Institute Inc., http://www.sas.com).

## Results

### Morphological analysis of *osgi* mutant rice plants

**Figure 1:**
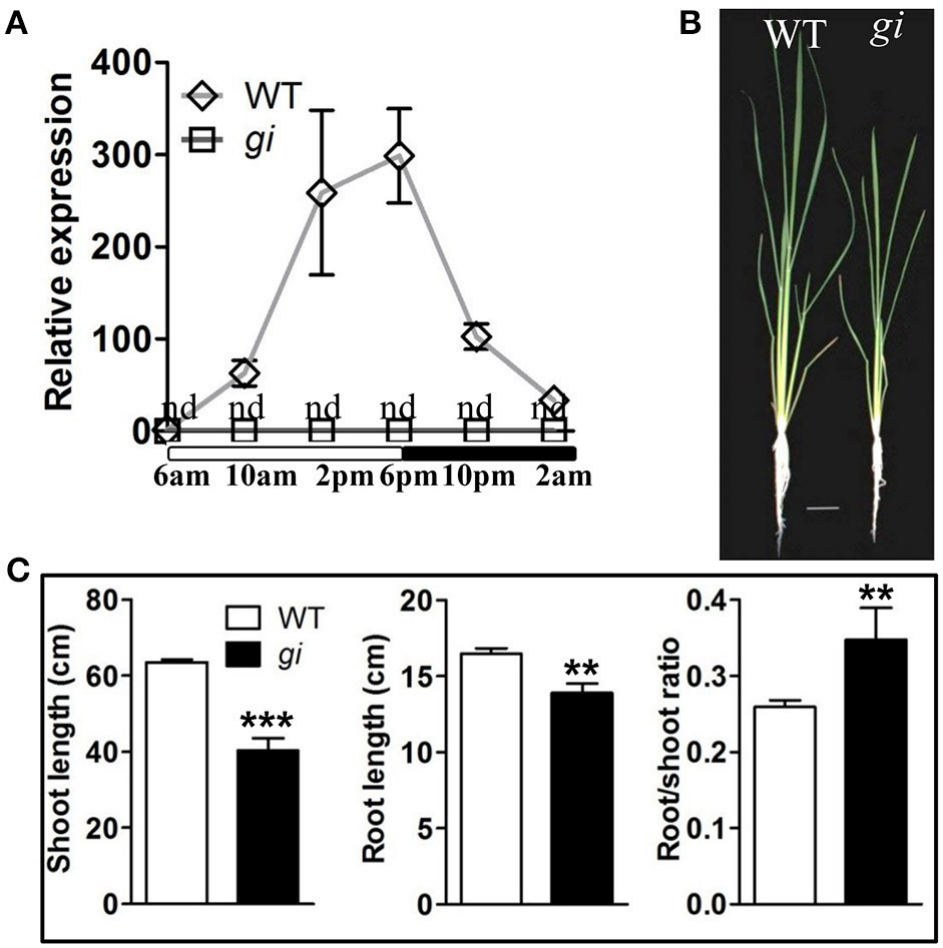
**Characterization of the growth phenotype of WT and ***osgi*** plants. (A)** Expression of *OsGI* in WT and *osgi (gi)* plants throughout the day. Unfilled and filled bars below the x-axis indicate times of lights-on and lights-off, respectively. Gene expression was normalized to the expression level of *OsACTIN*. **(B)** WT and *osgi* plants were grown hydroponically under normal growth conditions for 30 days. **(C)** Shoot length, root length and root/shoot ratio of WT and *osgi* plants. All data represent the mean of three biological replicates, with error bars indicating SD. Significant differences relative to the corresponding WT strain are indicated with asterisks (^***^P < 0.001; and ^**^P < 0.01). Bars = 4 cm. nd, not detectable.

The *TOS17* insertion mutant of *osgi* was obtained and characterized. As shown in Figure 1, the cyclic expression pattern of *OsGI*, which peaks at dusk in wild type (WT) plants, was completely abolished in *osgi* mutant plants (Figure 1A). Circadian rhythm genes, such as *OsRFT1* and *OsHd3a*, were also suppressed in *osgi* plants (Supplementary Figure 1). *Osgi* mutants displayed a growth inhibited phenotype (Figure 1B; Itoh and Izawa, 2011). Shoot and root lengths of 30-day-old *osgi* plants were reduced by 35 and 15% compared to WT plants, respectively, resulting in an increased root-to-shoot ratio (Figure 1C).

**Figure 2:**
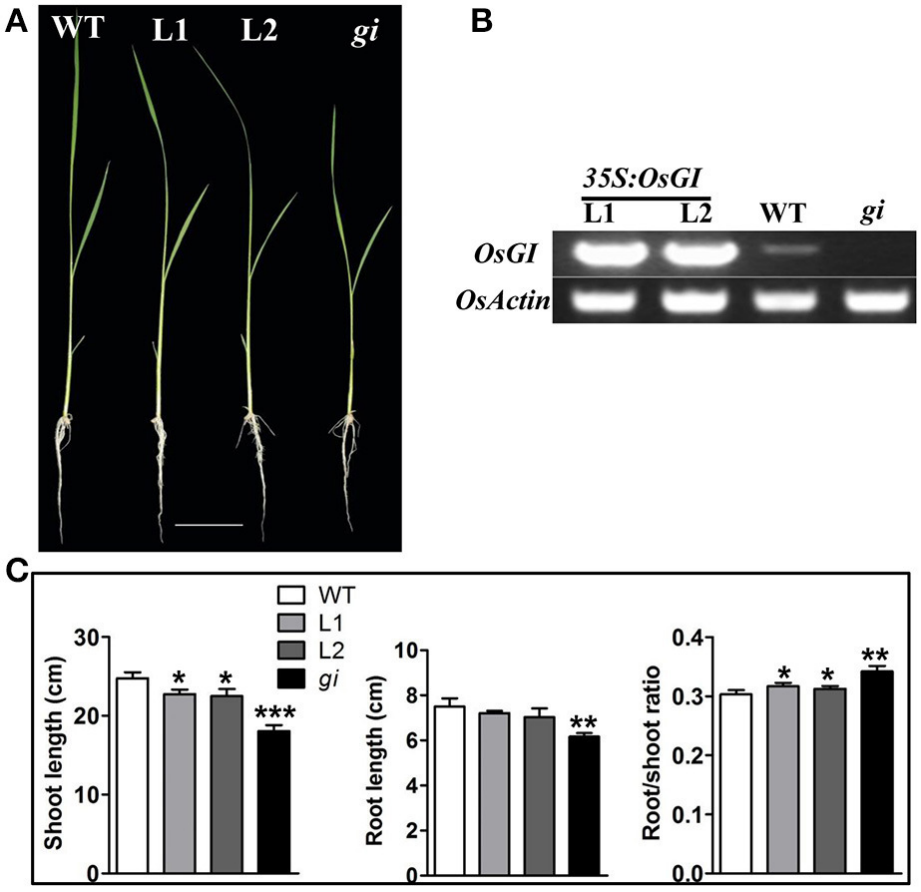
**Characterization of the growth phenotype in ***osgi***-complemented plants. (A)** WT, *osgi* mutant (*gi*) and two *OsGI*-complemented *osgi* mutant lines (L1 and L2) grown hydroponically under normal conditions for 14 days. **(B)** *OsGI* expression in WT, *osgi*, L1 or L2 plants 4 h after lights-on measured by RT-PCR. Plants were grown under normal conditions for 14 days. **(C)** Comparison of shoot length, root length and root/shoot ratio of WT, L1, L2 and *osgi* plants. All data represent the mean of three biological replicates, with error bars indicating SD. Significant differences relative to the corresponding WT strain are indicated with asterisks (^***^P < 0.001; ^**^P < 0.01; and ^*^P < 0.05). Bars = 4 cm.

To confirm that the growth defects were caused by the mutation in *OsGI*, genetic complementation was carried out by introducing the *OsGI* coding sequence under control of the CaMV35S promoter into *osgi* mutants in two transgenic events, L1 and L2. As shown in Figure 2A, the overexpression of *OsGI* in the *osgi* background rescued the growth defect observed in the mutant. The relative transcript abundance of *OsGI*, measured by reverse-transcription PCR (RT-PCR), was significantly higher in L1 and L2 than in either WT or *osgi* plants (Figure 2B, Supplementary Figure 2). Compared to *osgi* plants, the root and shoot lengths were increased in L1 and L2 while the root-to-shoot ratio decreased (Figure 2C). Based on these results, the L1 and L2 plants were used as overexpression lines in later experiments.

### Mutation of *OsGI* improved plant osmotic stress tolerance

**Figure 3:**
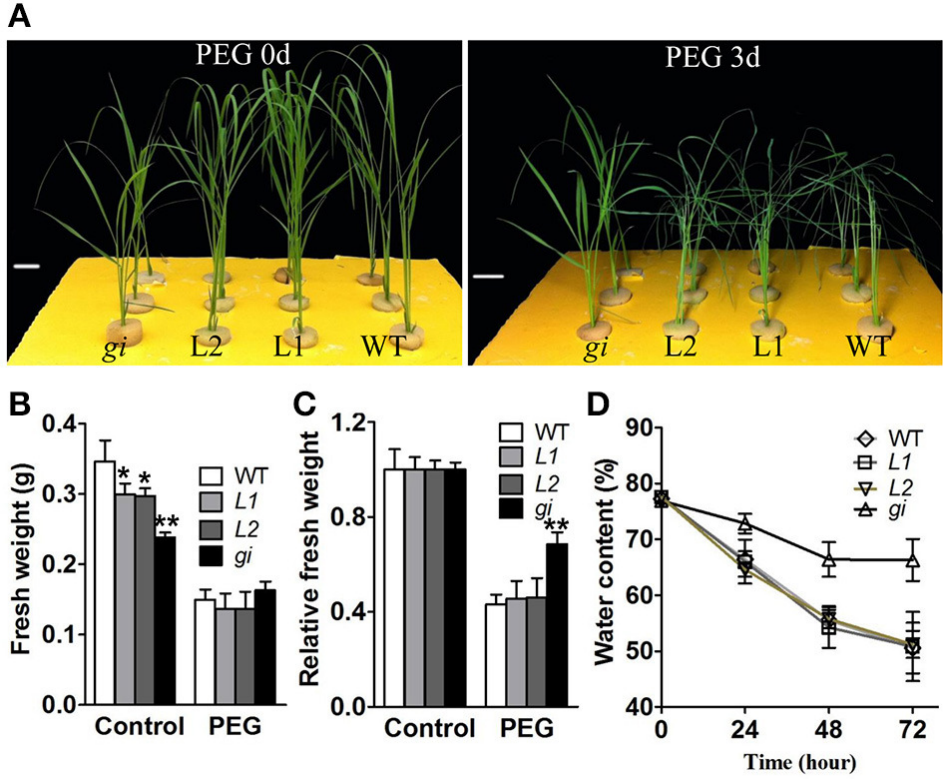
**Growth characteristics of WT, L1, L2 and ***osgi*** plants under PEG-treated conditions. (A)** 15-day-old WT, L1, L2 and *osgi* seedlings (left) were treated in 21% PEG-conditioned culture media for 3 days (right). **(B)** Absolute fresh weight or **(C)** fresh weight relative to the strain-matched control of WT, L1, L2 and *osgi* plants grown in control or PEG-treated culture media. **(D)** Water contents in WT, L1, L2 and *osgi* leaves were measured 24, 48, and 72 h after PEG treatment. All data represent the mean of three biological replicates, with error bars indicating SD. Significant differences relative to the corresponding WT strain are indicated with asterisks (^**^P < 0.01; and ^*^P < 0.05). Bars = 4 cm.

*OsGI* has been reported to be a molecular switch connecting flowering time regulation with multiple stress-response pathways, such as oxidative and salinity stresses (Kurepa et al., 1998; Kim et al., 2013). To investigate whether GI influences the tolerance to osmotic stress, WT and *osgi* plants were grown for 15 days under normal growth condition and then treated with 21% PEG 8000 for 3 days. As shown in Figure 3A, *osgi* mutants displayed improved osmotic stress tolerance compared to WT. After 3 days of exposure to high concentrations of PEG, WT plants were completely wilted while *osgi* plants remained healthy. PEG treatment decreased the fresh weight of WT and *osgi* plants by 55 and 30%, respectively, compared to the corresponding plants grown under normal condition (Figures 3B,C). While the water content in both WT and *osgi* leaves decreased during PEG treatment, *osgi* plants maintained a significantly higher water content than WT (Figure 3D). However, *osgi* plants used for the analysis of resistance to drought in soil showed the same phenotype as WT (data not shown).

**Figure 4:**
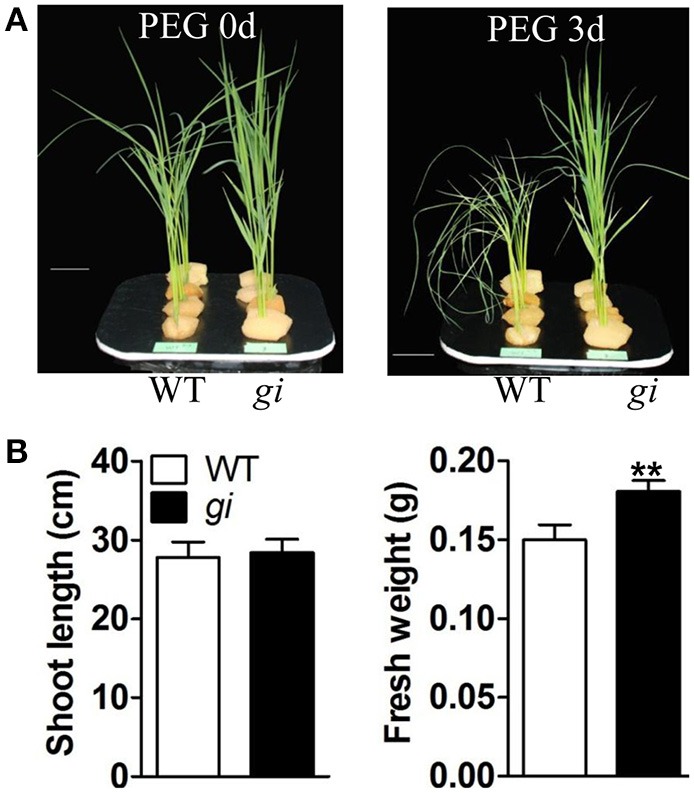
**Growth characteristics of size-matched WT and ***osgi*** plants in response to PEG treatment**. **(A)** 11-day-old WT and 14-day-old *osgi* seedlings, displaying the same plant height, were treated with 21% PEG condition for 3 days. **(B)** Shoot length and fresh weight of WT and *osgi* plants in (**A**). All data represent the mean of three biological replicates with error bars indicating SD. Significant differences relative to the corresponding WT strain are indicated with asterisks (^**^P < 0.01). Bars = 4 cm.

To exclude the possibility that the improved osmotic stress tolerance in *osgi* plants was due to its relatively smaller size (Figures 1B, 3A), additional osmotic stress tests were performed using size-matched WT plants and *osgi* seedlings at 11 DAG (WT) and 14 DAG (*osgi*), respectively (Figures 4A,B). Results showed that *osgi* plants still exhibited much higher tolerance to PEG treatment than WT plants (Figure 4A).

To confirm these results, the *OsGI* overexpression lines L1 and L2 were analyzed for their tolerance to osmotic stress. Results showed that L1 and L2 lines displayed increased sensitivity to osmotic stress under PEG exposure (Figure 3) compared to *osgi* mutants. However, we did not detect any differences between WT and *OsGI* overexpressing plants in response to PEG treatment (Figure 3). These results confirmed that *OsGI* functions as a negative factor in the osmotic tolerance of rice.

### Mutation of *OsGI* increases proline and sucrose contents in rice

**Figure 5:**
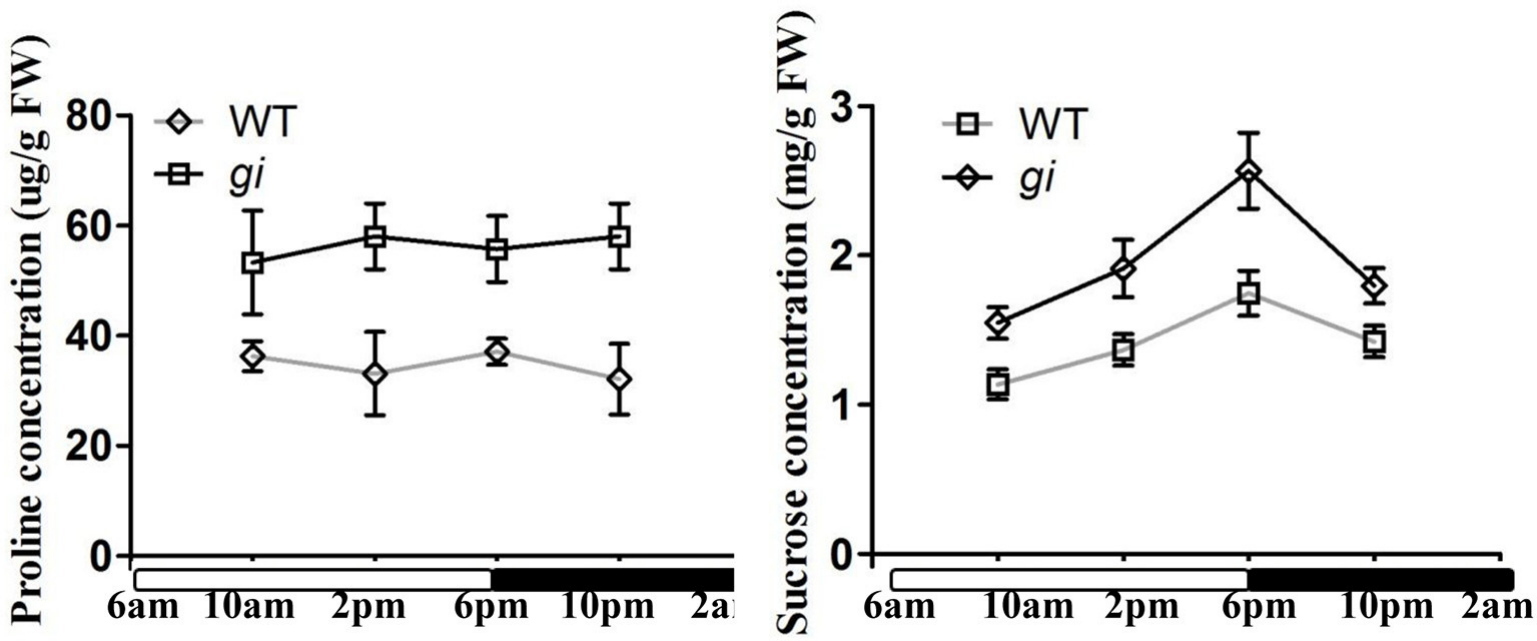
**Analysis of proline and sucrose contents in WT and ***osgi*** plants**. Proline and sucrose contents in leaves of 15-day-old WT and *osgi* plants grown under normal conditions. All data represent the mean of three biological replicates, with error bars indicating SD. The unfilled and filled bars below the x-axis indicate times of lights-on and lights-off, respectively.

It is generally accepted that under abiotic stress conditions, plants often accumulate compatible osmolytes to maintain osmotic balance and protect their subcellular structures from damage. Several studies have shown that the accumulation of free proline is positively correlated to plant tolerance to dehydration stress (Shinozaki and Yamaguchi-Shinozaki, 2007; Xiong et al., 2012). In order to assess whether mutation of *OsGI* can enhance the accumulation of osmotic protectants, the free proline content in WT and *osgi* plants was analyzed (Figure 5). Leaves from plants grown under normal conditions were collected at four different time points at 15 DAG and the proline content measured. At all times, the proline content was higher in *osgi* than in WT plants. For example, 8 h after lights-on, the proline levels in *osgi* plants were approximately 1.7 times higher than in WT plants (Figure 5). Moreover, while sucrose levels increased throughout the day and decreased at night in both WT and *osgi* plants, *osgi* plants had higher sucrose levels than WT plants at all times (Figure 5). The sucrose content in L1 and L2 plants was not altered compared to WT plants (Supplementary Figure 3). These results suggest that *osgi* plants possess a much higher osmotic potential, which could be beneficial in protecting plants against osmotic stress.

### Stomata movement in WT and *osgi* plants

In Arabidopsis, *GI* has been shown to be involved in the regulation of stomata opening (Ando et al., 2013). To investigate whether *OsGI* participates in the regulation of stomata movement under dehydration stress conditions, the stomata conductance (g_s_) and transpiration rate (T_r_) in WT and *osgi* plants were measured using LI-6400. 35-day-old WT and *osgi* seedlings, grown under normal conditions or treated with 18% PEG 8000 at 4, 6, and 8 h after lights-on, were used for the experiment. The g_*s*_ and T_*r*_ were recorded 9 h after lights-on (1, 3, and 5 h after PEG treatment, respectively) using an Li-6400.

**Figure 6:**
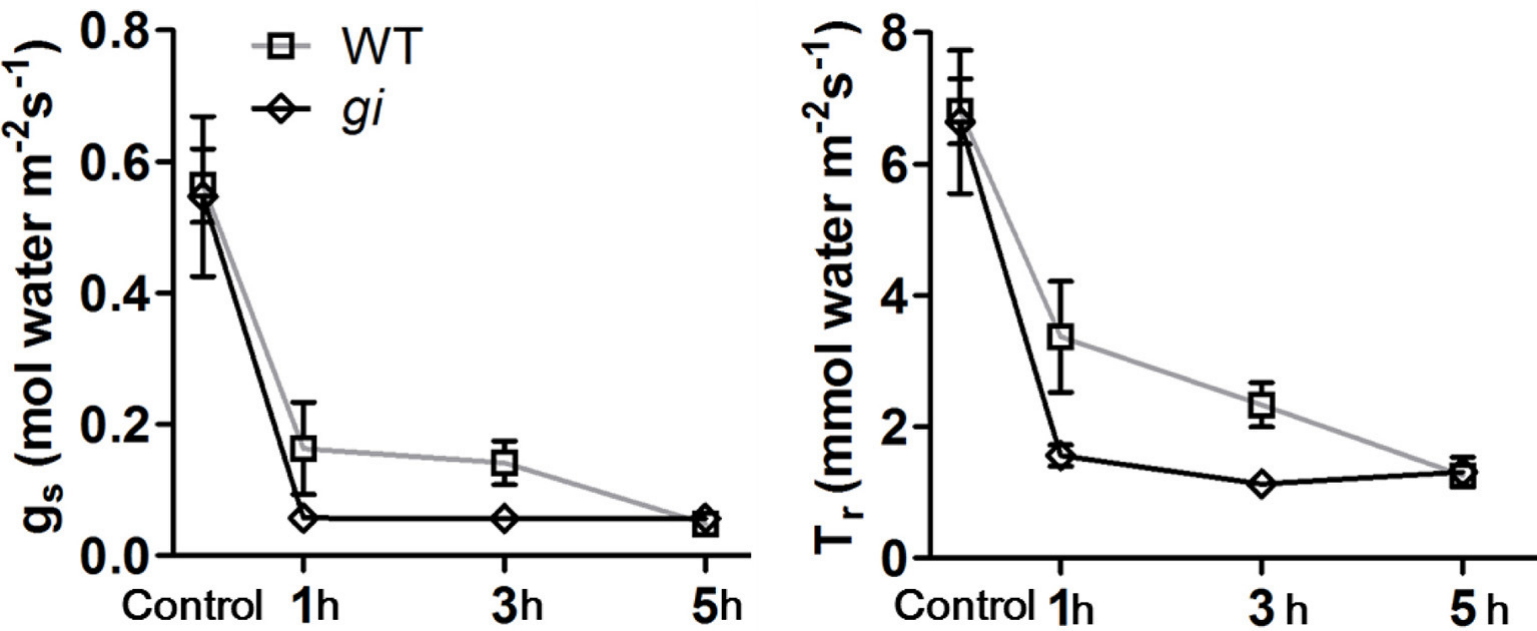
**Analysis of stomata conductance (gs) and transpiration rate (Tr) in WT and ***osgi*** plants**. Stomata conductance (gs) and transpiration rate (Tr) in WT and *osgi* plants after PEG treatment for 1, 3, and 5 h. 35-day-old WT and *osgi* plants grown under normal conditions or treated with 18% PEG 8000 at 4, 6, and 8 h after lights-on, respectively, were used for the experiment. The g_s_ and T_r_ were recorded in *osgi* and WT plants using an Li-6400 at 9 h after lights-on. All data represent the mean of three biological replicates, with error bars indicating SD.

Under normal growth conditions, there was no difference in the g_*s*_ and T_*r*_between WT and *osgi* plants (Figure 6). PEG treatment reduced the g_s_ and T_r_ in both WT and *osgi* plants. The g_*s*_ decreased by 70, 75, and 90% in WT plants after PEG treatment for 1, 3, and 5 h, respectively. In contrast, the g_*s*_ in *osgi* seedlings decreased rapidly by about 90% after only 1 h of PEG treatment and stayed at a similarly low level after 3 and 5 h of PEG exposure. The change in T_r_ in response to PEG treatment followed a similar pattern as g_*s*_ (Figure 6). These results indicate that under osmotic stress conditions, stomata movement changes faster in *osgi* mutant plants than in WT plants.

### Mutation of *OsGI* alters the expression pattern of dehydration-related genes

**Figure 7:**
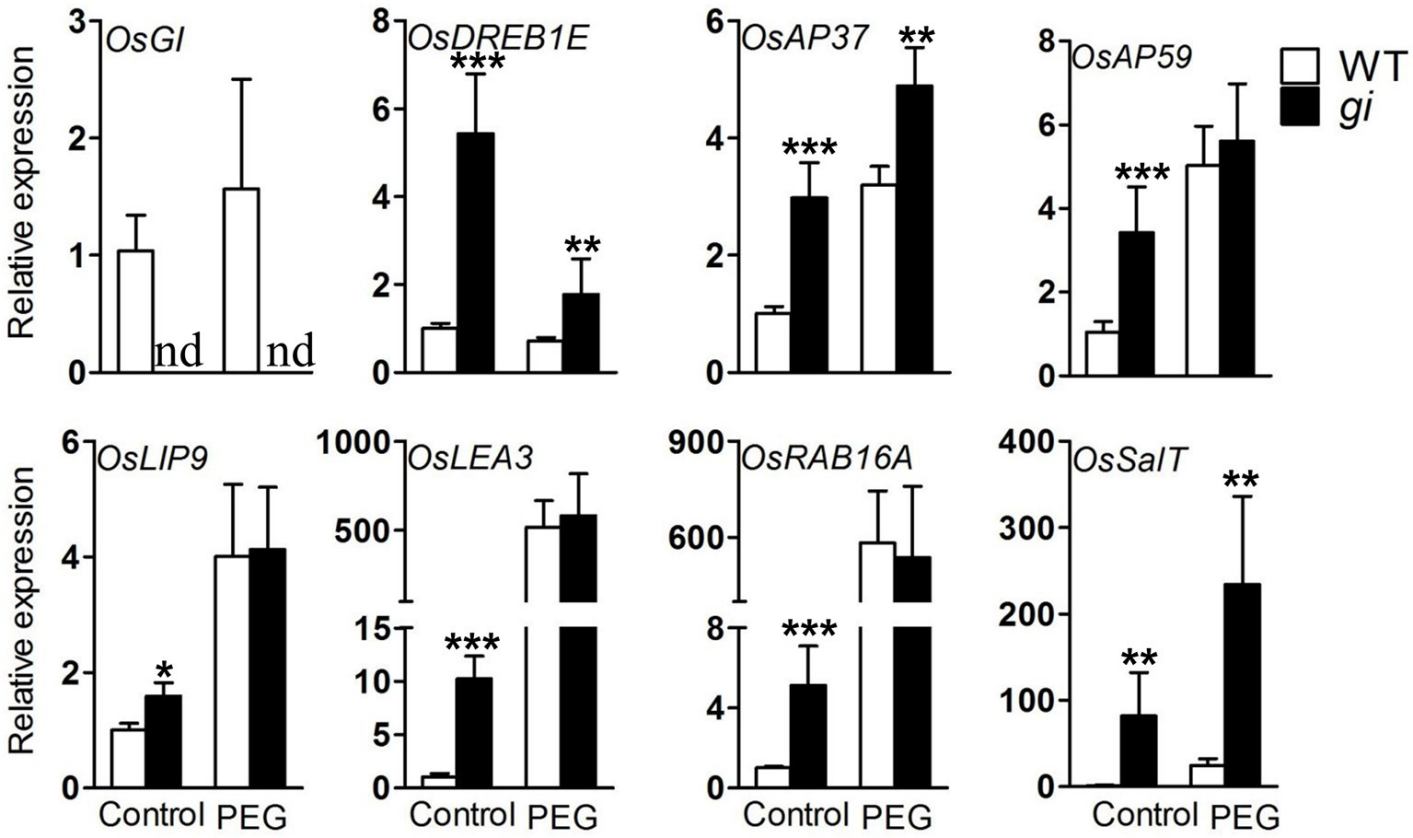
**Expression of water deficiency-related genes in WT and ***osgi*** leaves**. WT and *osgi* plants were grown under normal conditions for 15 days and then cultured in 18% PEG 8000 or normal culture media for 2 days. Leaves were sampled 8 h after lights-on. Total RNA was extracted from the leaves and used for qRT-PCR. All data represent the mean of three biological replicates, with error bars indicating SD. Expression of *OsACTIN* was used as the internal control. Significant differences relative to the corresponding WT strain are indicated with asterisks (^***^P < 0.001; ^**^P < 0.01; and ^*^P < 0.05).

To investigate the impact of *OsGI* mutation on the expression of genes involved in the response to dehydration stress, qRT-PCR was carried out on leaf samples of WT and *osgi* plants grown under normal or PEG treatment conditions. The expression of *OsGI* was not affected by osmotic stress (Figure 7, Supplementary Figure 4). *OsDREB1E, OsAP37, OsAP59, OsLEA3, OsRAB16A, OsLIP9*, and *OsSalT*, which have been shown to be positively associated with osmotic stress tolerance, were selected for analysis (Chaves et al., 2003; Umezawa et al., 2006; Valliyodan and Nguyen, 2006; Shinozaki and Yamaguchi-Shinozaki, 2007; Xiao et al., 2007; Fukao et al., 2011). As expected, the expression of *OsAP37, OsAP59, OsLEA3, OsRAB16A, OsLIP9*, and *OsSalT* were induced by osmotic stress (Figure 7). Under normal conditions, the transcript abundance of all tested genes was significantly higher in *osgi* mutants than in the WT. Osmotic stress conditions further increased the expression of *OsAP37, OsAP59*, and *OsSalT* in *osgi* mutants (Figure 7). These results suggest that dehydration-responsive genes are constitutively active in *osgi* mutant plants.

**Figure 8:**
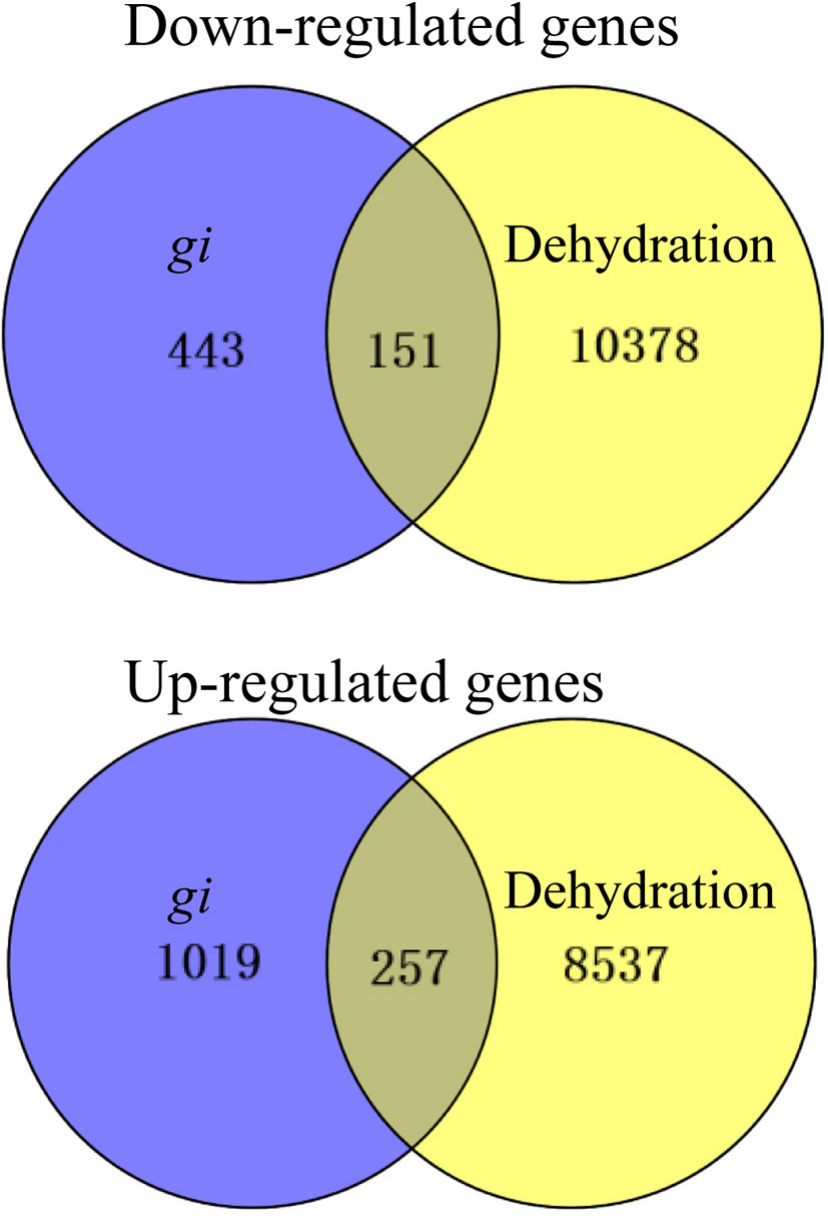
**Transcriptomic analyses of ***osgi*** and WT seedlings**. Venn diagram representing the number of differentially expressed genes in *osgi* seedlings compared to WT seedlings grown under normal conditions (top; *p* < 0.05, PPDE > 0.95, 2-fold cut-off) or to WT seedlings exposed to dehydration conditions (bottom; Jain et al., 2007). For the microarray, rice seedlings were grown for 14 days under normal conditions before leaves were harvested.

To elucidate the molecular function of *OsGI*, the expression profiles in *osgi* mutant and WT leaves were analyzed using Affymetrix GeneChip. Microarray analysis revealed that mutation of *osgi* results in substantial transcriptomic reprogramming. Indeed, 1870 genes were differentially expressed in the *osgi* mutant background compared to WT plants (*p* < 0.05, PPDE > 0.95, 2-fold cut-off), of which 594 and 1267 genes were down- or up-regulated, respectively (Figure 8). Given the increased osmotic stress tolerance in *osgi* plants, we compared the mutant transcriptome to a dehydration-altered transcriptome of WT rice (Qian et al., 2014). In summary, 151 down-regulated and 257 up-regulated genes in *osgi* overlapped with the reported dehydration stress responses (Figure 8; Jain et al., 2007).

In order to further assess how *OsGI* mutation alters dehydration stress-related genes, we analyzed the expression pattern of water deficiency-related genes in leaves in our microarray. Abscisic acid (ABA) is thought to be involved in the regulation of stomata movement and plays a critical role in the response to drought (Chaves et al., 2003). Given the regulation of *OsGI* in stomata opening, the expression of ABA-related genes was examined in *osgi* plants (Table 1). A number of genes involved in ABA signaling displayed altered expression in *osgi* leaves, including several ABA-induced proteins and protein phosphatase 2C (PP2C) genes in subfamily A (Table 1; Schweighofer et al., 2004). Since improved antioxidant capacity is critical in order for plants to survive in water-limited conditions, the expression levels of antioxidant genes were investigated in the microarray (Mittler et al., 2004; Hu et al., 2008). Surprisingly, mutation of *OsGI* activated antioxidant genes including superoxide dismutase, peroxidase and thioredoxin (Table 2), indicating that *osgi* plants are strong Reactive Oxygen Species (ROS) scavengers. Furthermore, increased expression of chaperones which protect proteins from stress damage has been shown to improve plant tolerance to water deficits (Wang et al., 2004). Accordingly, the expression of various chaperone genes such as heat shock proteins increased in *osgi* plants (Table 3).

**Table 1:** **ABA signaling-related genes with significantly altered transcript abundance in ***osgi*** compared to WT leaves (***p*** < 0.05)**.

**TIGR locus identifier**	**Fold change**	**Description**
	***osgi* vs. WT**	
**ABA**		
Os04g52090	2.54	OsAP2-39
Os03g09170	2.63	DRE binding factor
Os09g35010	2.11	HvCBF4
Os04g46440	3.23	DREB1C
Os11g30500	3.73	ABA induced protein
Os04g34600	1.58	ABA induced protein
Os06g14370	4.91	ABA induced protein
Os11g06720	1.63	Abscisic stress ripening protein (OsASR5)
Os07g07050	3.26	Abscisic-aldehyde oxidase
Os07g18120	1.75	Abscisic-aldehyde oxidase
Os02g52780	1.53	bZIP transcription factor ABI5
Os05g50800	4.22	ABIL1 protein
Os02g03960	−2.52	Transcription factor,OsbZIP14
Os02g49860	−11.10	ABA induced plasma membrane protein PM 19
Os02g50140	−2.95	ABA-induced protein
Os09g07380	−1.35	ABI3-interacting protein 2
Os01g73250	−3.29	Abscisic stress-ripening (OsASR6)
**PP2C**		
Os01g40094	3.98	Protein phosphatase 2C
Os05g46040	1.47	Protein phosphatase 2C
Os03g16170	4.06	Protein phosphatase 2C
Os09g15670	7.29	Protein phosphatase 2C
Os01g62760	1.55	Protein phosphatase 2C
Os01g46760	1.52	Protein phosphatase 2C

*TIGR locus identifiers are given for each transcript at the left. Expression ratio in osgi vs. WT leaves is shown. The annotation description of each gene is according to the TIGR and RAP general description. Positive numbers indicate increased gene expression whereas negative numbers indicate decreased gene expression*.

**Table 2:** **Genes involved in antioxidant synthesis with significantly altered transcript abundance in ***osgi*** compared to WT leaves (***p*** < 0.05)**.

**TIGR locus identifier**	**Fold change**	**Description**
	***osgi* vs. WT**	
Os07g46990	1.59	Superoxide dismutase
Os08g44770	1.92	Superoxide dismutase
Os06g02500	2.21	Superoxide dismutase
Os05g25850	1.29	Superoxide dismutase
Os01g61320	2.19	Thioredoxin family protein
Os03g58630	1.24	Thioredoxin H-type 5 (OsTrx10)
Os01g07376	1.65	Thioredoxin H-type (OsTrx1)
Os02g56900	2.31	Thioredoxin family protein
Os08g29110	1.44	Thioredoxin family protein
Os02g53400	1.30	Thioredoxin-like 5, chloroplast precursor
Os05g11090	1.45	Thioredoxin-like 6, chloroplast precursor
Os04g15690	5.24	DSBA-like thioredoxin domain containing protein
Os06g37080	1.55	L-ascorbate oxidase precursor
Os11g42220	4.06	L-ascorbate oxidase precursor
Os08g44340	2.50	monodehydroascorbate reductase
Os07g49400	1.19	OsAPx2—Cytosolic Ascorbate Peroxidase gene
Os01g19020	15.16	Peroxidase 1 precursor
Os05g04380	18.38	Peroxidase 1 precursor
Os01g22370	1.62	Peroxidase 1 precursor
Os01g22230	2.37	Peroxidase 1 precursor
Os10g39170	1.33	Peroxidase 1 precursor
Os01g07770	2.62	Peroxidase 25 precursor
Os06g46799	2.18	Peroxidase 39 precursor
Os05g04500	9.58	Peroxidase 63 precursor
Os06g13050	1.50	Peroxidase family protein
Os06g09610	2.64	Peroxiredoxin bcp
Os02g09940	5.88	Peroxiredoxin-5, mitochondrial precursor
Os01g16152	1.77	Peroxiredoxin-5, mitochondrial precursor
Os02g33450	1.67	2-cys peroxiredoxin BAS1, chloroplast precursor

**Table 3:** **Genes involved in chaperone synthesis with significantly different transcript abundance in ***osgi*** vs. WT leaves (***p*** < 0.05)**.

**TIGR locus identifier**	**Fold change**	**Description**
	***osgi* vs. WT**	
Os02g52150	2.93	Heat shock protein (OsHSP20)
Os06g11610	3.57	Heat shock protein
Os09g31486	2.20	Heat shock 70 kDa protein, mitochondrial precursor
Os02g53420	1.31	Heat shock 70 kDa protein, mitochondrial precursor
Os03g60620	1.25	Heat shock cognate 70 kDa protein 2
Os01g62290	2.36	Heat shock cognate 70 kDa protein
Os05g38530	2.25	Heat shock cognate 70 kDa protein
Os06g35960	3.03	Heat shock factor protein
Os04g48030	2.05	Heat shock factor protein
Os09g28354	4.39	Heat shock factor protein
Os01g43590	2.04	Heat shock factor protein HSF8
Os08g39140	1.30	Heat shock protein 81-1
Os03g18200	1.30	Heat shock protein DnaJ
Os12g31460	1.50	Heat shock protein DnaJ
Os01g53220	2.13	Heat shock factor protein (OsHsfC1b)
Os03g16040	1.84	Heat shock protein (OsHsp17.7)
Os02g54140	2.82	Heat shock protein
Os10g32550	1.99	Chaperonin CPN60-1, mitochondrial precursor
Os03g04970	1.85	Chaperonin CPN60-1, mitochondrial precursor
Os05g46290	1.69	Chaperonin CPN60-2, mitochondrial precursor
Os06g09679	1.29	Chaperonin, chloroplast precursor
Os09g26730	1.54	Chaperonin, chloroplast precursor
Os03g25050	2.18	Chaperonin
Os07g44740	1.78	Chaperonin
Os03g25050	1.79	Chaperonin

## Discussion

In the past decade, the identification of many key factors involved in the response to water deficits has greatly increased our understanding of molecular mechanisms participating in the osmotic response in plants. In our current study, we investigated the role of a key flowering time regulator, OsGI, in osmotic stress tolerance in rice. Our data revealed that *OsGI* plays a negative role in the rice response to water deficiency by regulating at both physiological and transcriptional levels. Results from our analysis suggest that in *osgi* mutant plants, several components of the osmotic stress response are constitutively activated.

Alteration of *OsGI* could have improved the plant osmotic stress response in two ways. One, the osmotic potential in the *osgi* mutant could have increased due to the constitutively altered morphology, metabolism and gene expression which led to the accumulation of osmoprotectants such as sucrose and proline. It has been shown that increased sucrose and proline contents improve plant tolerance to osmotic stress (Hadiarto and Tran, 2011). In *osgi* seedlings, concentrations of sucrose and proline increased in the leaves (Figure 5), which could have increased the plants' osmotic potential to defend against dehydration. Alteration of *OsGI* could also play a role in the osmotic stress response because rapid physiological adjustment has been shown to decrease water loss and improve water utilization (Hirayama and Shinozaki, 2010; Hadiarto and Tran, 2011; Xiong et al., 2012). In plants, water loss mainly occurs in transpiration via stomata opening. Stomata movement is critical for plants to reduce transpiration and to rapidly respond to osmotic stress conditions (Chaves et al., 2003; Valliyodan and Nguyen, 2006). In *osgi* plants, the accelerated stomata closure could have greatly improved water utilization by rapidly decreasing the transpiration rate in leaves, resulting in decreased water loss (Figures 3D, 6).

Although GI shares no known functional domains with other proteins, it has been shown to function in many pathways at the protein level (Shi et al., 2000; Kim et al., 2013; Park et al., 2013). In our study, expression of *OsGI* is not affected by osmotic stress at the transcriptional level (Figure 7, Supplementary Figure 4), indicating that OsGI might function purely on a protein level in the response to dehydration stress. Further study to determine the OsGI protein abundance under osmotic stress conditions will provide more insight into the molecular mechanisms by which OsGI responds to dehydration stress. Interestingly, mutation of *OsGI* resulted in substantial transcriptomic reprogramming, and 151 of the down- and 257 of the up-regulated genes in *osgi* leaves showed the same (down/up-regulated) response in WT leaves exposed to dehydration conditions (Figure 8). However, further studies are required to determine the mechanisms by which *OsGI* regulates the transcriptional response to osmotic pressure.

ABA is required for plants to adapt stomata movement to water-deficient conditions (Fujita et al., 2011). Because *OsGI* is involved in regulating stomata opening (Figure 6), ABA-related genes were examined in *osgi* plants. Results showed that a number of genes involved in ABA signaling were altered in *osgi* leaves (Table 1). Although the growth phenotype of rice *osgi* plants under ABA treatment was comparable to that of WT plants (data not shown), *GI* has been shown to function in ABA metabolism (Penfield and Hall, 2009; Riboni et al., 2013). Indeed, *GI* has been suggested to play a role in drought response and seed dormancy in an ABA-dependent pathway (Penfield and Hall, 2009; Riboni et al., 2013). *GI* serves as a core gene in the circadian rhythm of plants, and recent research revealed that around 40% of ABA-related genes are controlled by the circadian clock (Covington et al., 2008; Legnaioli et al., 2009). The circadian gene TOC1 participates in ABA signaling by regulating ABA-Related gene (ABAR) and by interacting with Abscisic Acid Insensitive 3 (ABI3) and is degraded by ZEITLUPE (ZTL) (Kurup et al., 2000; Kim et al., 2007). It is possible that GI functions in ABA signaling via TOC1 regulation by directly interacting with ZTL (Kim et al., 2007). In addition, the GI-CO-FT flowering time regulatory system is also conserved in stomata regulation (Ando et al., 2013). FT is required for maintaining H^+^-ATPase activation to regulate stomata opening (Kinoshita et al., 2011). GI might therefore be involved in the H^+^-ATPase-dependent regulation of stomata movement. Our results suggest that GI could regulate stomata movement via multiple pathways but how GI regulates stomata movement in response to osmotic stress still needs further investigation.

ROS can quickly accumulate in plants under osmotic stress and cause oxidative damage to proteins. As a result, ROS scavenging is critical for plants in order to survive under water deficient condition (Umezawa et al., 2006). In Arabidopsis, increased levels of superoxide dismutase and ascorbate peroxidase confer oxidative stress tolerance in *Atgi* plants (Kurepa et al., 1998; Cao et al., 2006). In rice, superoxide dismutase, peroxidase and thioredoxin are constitutively active in *osgi* plants to defend against oxidative damage (Table 2). Moreover, molecular chaperones are key factors in protein stabilization, and the increased expression of chaperone genes in *osgi* leaves is speculated to protect proteins from dehydration damage (Table 3; Wang et al., 2004).

Compared to WT, a majority of the alterations in *osgi* plants, such as sucrose, were observed both in the field and greenhouse (Figure 5; Izawa et al., 2011). However, transcriptome and metabolome analysis revealed that *osgi* plants exhibited different responses in the field and greenhouse in some pathways. While stomata conductance was higher in field-grown *osgi* than WT plants, there was no difference between *osgi* and WT plants grown under normal conditions in the greenhouse (Figure 6; Izawa et al., 2011). Conversely, *osgi* plants grown in the greenhouse had higher proline contents than WT plants (Figure 5) while there was no difference in the proline contents of field-grown *osgi* and WT plants (Izawa et al., 2011). The TCA cycle intermediate malate was decreased in field-grown but increased in greenhouse-grown *osgi* plants (Izawa et al., 2011). In addition, Expressions of some key genes in the phenylpropanoid pathway, a secondary metabolite pathway controlled by circadian clocks, was significantly upregulated in *osgi* plants in the field, but none of these genes were altered in our microarray, indicating that OsGI function is affected by the environment (Izawa et al., 2011).

The tolerance to osmotic stress prompted us to check whether *osgi* plants exhibit drought resistance but we did not detect any differences between WT and *osgi* plants grown in soil under drought condition (data not shown), nor did we find any differences between WT and *osgi* plants in response to salt stress. In Arabidopsis, GI-deficient plants exhibit increased sensitivity to drought but improved tolerance to salt stress (Han et al., 2013; Kim et al., 2013), suggesting divergent functions of GI in drought and salt resistance in rice and Arabidopsis. Seedlings encounter sudden dehydration stress when transferred from normal culture media to PEG-treated media, as opposed to soil-grown plants where water depletion and thus drought formation is much more gradual, commonly occurring over hours or even days. In conclusion, while *osgi* plants are capable of tolerating sudden osmotic stress to some degree, further study is needed to better understand the mechanisms by which OsGI is involved in drought response.

In summary, we have shown that *OsGI* is an essential regulator of the plant response to dehydration stress, and modification of *OsGI* expression might improve the osmotic tolerance of crop cultures. However, more work remains to be done to fully understand the mechanisms of action by which *OsGI* functions in the regulation of the plant stress response.

## Author contributions

HS conceived and designed the research. SL, WY, MW, WQ, and LZ conducted the experiments and analyzed the data. SL and HS wrote the manuscript. All authors read and approved the manuscript.

### Conflict of interest statement

The authors declare that the research was conducted in the absence of any commercial or financial relationships that could be construed as a potential conflict of interest.
