# Supplementary material for: Mutation of OsGIGANTEA Leads to Enhanced Tolerance to Polyethylene Glycol-Generated Osmotic Stress in Rice
Source: Front Plant Sci. 2016 Apr 18;7:465. doi: 10.3389/fpls.2016.00465 (PMC4834575; doi:10.3389/fpls.2016.00465)
Supplement: Supplementary file 1 [file Table1.PDF]

Supplementary Material

Mutation of *OsGIGANTEA* leads to enhanced tolerance to polyethylene glycol-generated osmotic stress in rice

Shuai Li[1, 2], Wenhao Yue[1], Min Wang[1], Wenmin Qiu [1], Lian Zhou[1], Huixia Shou[1,*]

***Correspondence:** Huixia Shou: huixia@zju.edu.cn

Supplementary Table 1. Primer used in this study

Primers for identifying *osgi* mutant	
OsGI-F	AGGGCTAACGGTTCTCCAAG
OsGI-R	TTATGAGTTCCACCCCTTGC
TOS17	ATTGTTAGGTTGCAAGTTAGTTAAGA
Primers used for qRT-PCR or RT-PCR	
OsDREB1E-F	ACTTCCCTTGCTACCCGATG
OsDREB1E-R	GCTCCATAGATTGACCTCGCAG
OsAP37-F	TCCGATGTTTTGGTCCTCTG
OsAP37-R	TCCACGGTTTAGTCCATCTCATC
OsAP59-F	GGTGATTTAGCCATCTTGTGCG
OsAP59-R	TCGTCACATTTCTTGGAGCAG
OsRAB16A-F	CATGGACAAGATCAAGGAGAAGC
OsRAB16A-R	CTTATTATTCAGGAAGGTGACGTGG
OsLEA3-F	GCCGTGAATGATTTCCCTTTG
OsLEA3-R	CACACCCGTCAGAAATCCTCC
OsLIP9-F	TGGAATTTGGAAGTGTTTGGC
OsLIP9-R	CCCACACGAAACACAAACTTC
OsGI-RT-F	TGGAATGCTTGTTGATGGT
OsGI-RT-R	GCGGATGGTGGTTGATAGA
OsHd3a-RT-F	GCTAACGATGATCCCGAT
OsHd3a-RT-R	CCTGCAATGTATAGCATGC
OsRFT1-RT-F	CGGCTGGTGTTCGTGCTGTTC
OsRFT1-RT-R	TGGCAGTTGAAGTAGACGGTGG
OsACTIN-RT-F	CAACACCCCTGCTATGTACG
OsACTIN-RT-R	CATCACCAGAGTCCAACACAA

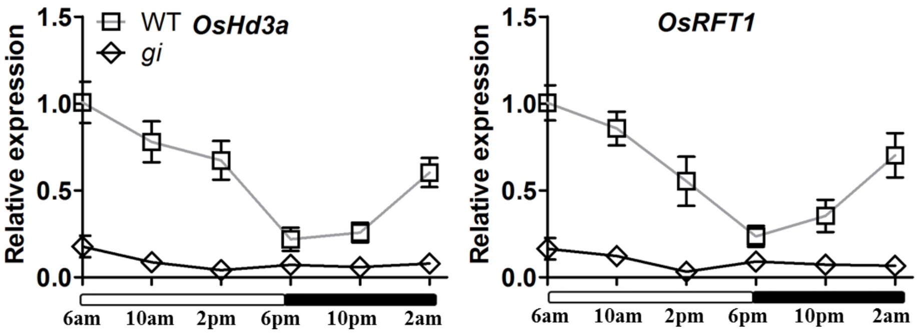

Supplementary Figure 1. Expressions of *OsHd3a* and *OsRFT1* in WT and *osgi* plants throughout the day. WT and *osgi* (*gi*) plants were grown hydroponically under normal growth conditions for 30 days. Unfilled and filled bars below the x-axis indicate times of lights-on and lights-off, respectively.

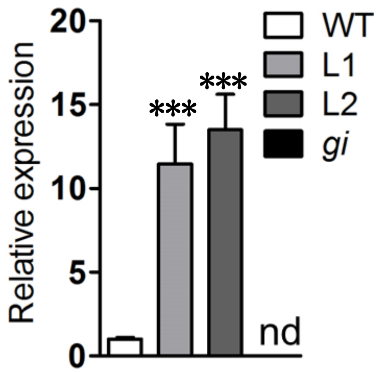

Supplementary Figure 2. *OsGI* expression in WT, *osgi*, L1 or L2 plants 4 hours after lights-on measured by qRT-PCR. L1, L2 means two complemented lines. Plants were grown in normal condition for 14 days, nd, not detected. *** is significantly different at P< 0.001, compared with the relative WT.

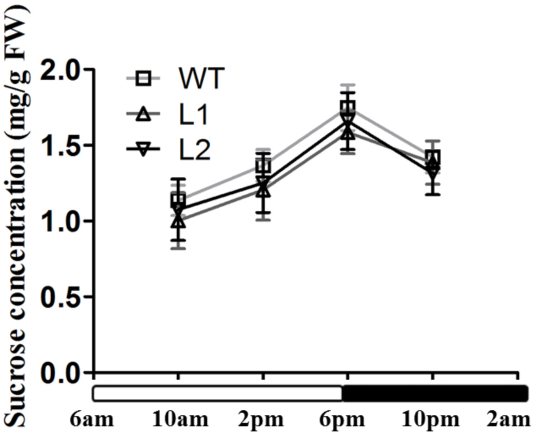

Supplementary Figure 3. Analysis of sucrose contents in WT, L1 and L2 plants. Sucrose contents in leaves of 15-day-old WT, L1 and L2 plants grown in normal condition were measured. All data represent the mean of three biological replicates, with error bars indicating SD. The unfilled and filled bars below the x-axis indicate

times of lights-on and lights-off, respectively.

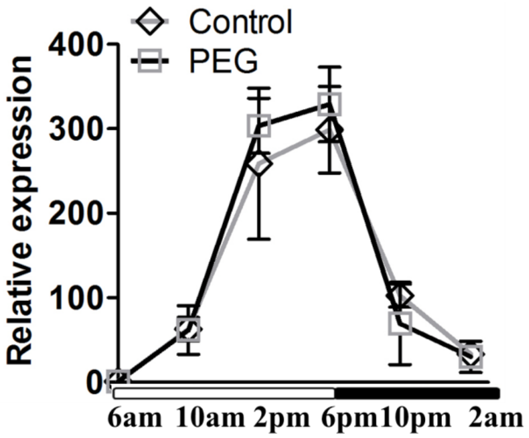

Supplementary Figure 4. Expression of *OsGI* in response to PEG treatment in WT plants throughout the day. The Unfilled and filled bars below the x-axis indicate times of lights-on and lights-off, respectively. WT plants were grown in normal conditions for 15 days, and then transferred to 18% PEG8000 treated or normal condition for 2 days and then leaves were sampled. Total RNA was extracted from the leaves and used for qRT-PCR. All data are means of three biological replicates with error bars indicating SD. Expression of *OsACTIN* was used as the internal control.
